# Amino Acid Sensing and Assimilation by the Fungal Pathogen *Candida albicans* in the Human Host

**DOI:** 10.3390/pathogens11010005

**Published:** 2021-12-22

**Authors:** Fitz Gerald S. Silao, Per O. Ljungdahl

**Affiliations:** Department of Molecular Biosciences, Wenner-Gren Institute, SciLifeLab, Stockholm University, 114 19 Stockholm, Sweden; fitzgerald.silao@scilifelab.se

**Keywords:** *Candida albicans*, human fungal pathogen, nutrient sensing, amino acid metabolism, proline catabolism, mitochondria, SPS-sensor, nitrogen catabolite repression, glucose repression

## Abstract

Nutrient uptake is essential for cellular life and the capacity to perceive extracellular nutrients is critical for coordinating their uptake and metabolism. Commensal fungal pathogens, e.g., *Candida albicans*, have evolved in close association with human hosts and are well-adapted to using diverse nutrients found in discrete host niches. Human cells that cannot synthesize all amino acids require the uptake of the “essential amino acids” to remain viable. Consistently, high levels of amino acids circulate in the blood. Host proteins are rich sources of amino acids but their use depends on proteases to cleave them into smaller peptides and free amino acids. *C. albicans* responds to extracellular amino acids by pleiotropically enhancing their uptake and derive energy from their catabolism to power opportunistic virulent growth. Studies using *Saccharomyces* *cerevisiae* have established paradigms to understand metabolic processes in *C. albicans*; however, fundamental differences exist. The advent of CRISPR/Cas9-based methods facilitate genetic analysis in *C. albicans*, and state-of-the-art molecular biological techniques are being applied to directly examine growth requirements in vivo and in situ in infected hosts. The combination of divergent approaches can illuminate the biological roles of individual cellular components. Here we discuss recent findings regarding nutrient sensing with a focus on amino acid uptake and metabolism, processes that underlie the virulence of *C. albicans*.

## 1. Introduction

All organisms require nutrients to live, grow and successfully reproduce. The ability of an organism to assimilate nutrients in a given ecological niche is dependent on its ability to sense and respond to the availability of nutrients and on intrinsic cellular properties. Defining the key signaling events activated by nutrient sensing systems and the metabolic capacities of an organism provides a compelling description that reflects the organism’s role in the niche. For opportunistic human pathogens, acquiring nutrients to support commensal or pathogenic growth is not a trivial task as the availability of key nutrients is dependent on several extrinsic host-specific factors. Such factors include host defense activities that often are linked to substantial and rapid changes in biophysical parameters, e.g., extracellular pH and the generation of reactive oxygen species (ROS), different nutrient and metabolic activities of host tissues at sites of fungal cell colonization, and the presence of competing microorganisms.

**Figure 1:**
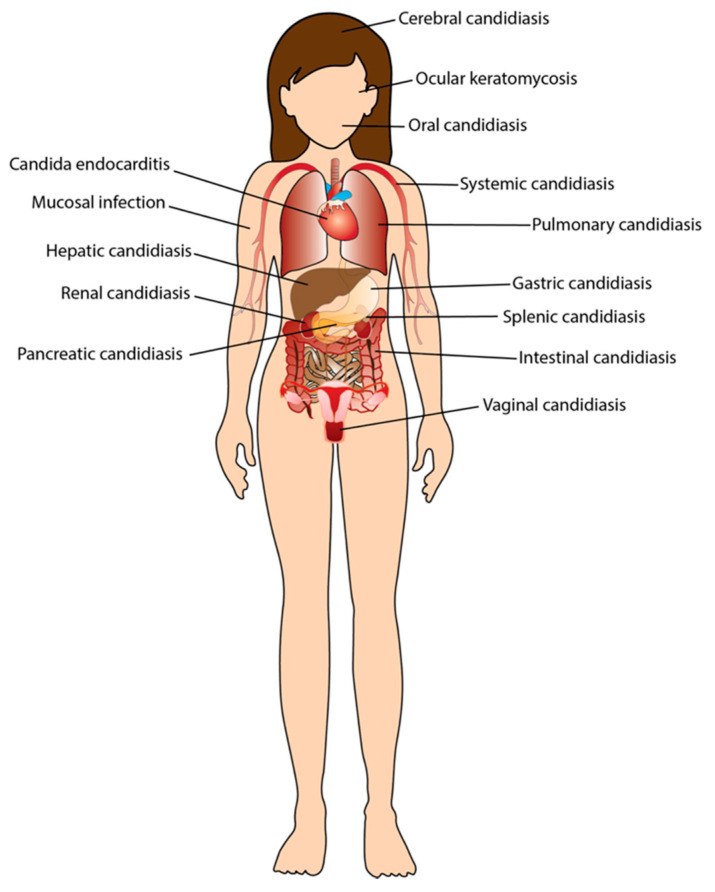
***C. albicans* can infect virtually all anatomical sites in the body.** Infections can either be superficial, mainly affecting the skin or mucous membrane, or invasive, involving fungal entry into the blood (candidemia) and dissemination to other internal organs. Disseminated growth happens when *C. albicans*, colonizing an anatomical site (usually the gut), catheters or other medical implants, enter the blood and then disseminate to other organs such as the lungs (pulmonary), liver (hepatic), spleen (splenic), pancreas (pancreatic) and kidney (renal). These infections can be localized or can re-enter the bloodstream again, allowing them to reach additional anatomical sites, and in rare cases the brain (cerebral). *C. albicans* in the blood can enter the urine via the kidney, resulting in candiduria (yeast in the urine). Other complications of *Candida* infections include the appearance of fungus balls in certain sites, resulting in obstruction in these areas and the formation of abscesses. In women, vaginal candidiasis is an important concern since the vagina serves as a main reservoir for *C. albicans*. In addition, infection of the genitourinary tract is more often diagnosed in women than in men due to anatomical structural differences such as a shorter urethra and the close proximity of the vagina and anus.

Of the fungal pathogens capable of infecting humans, *Candida albicans* is considered to be the most important, and arguably the most successful. *C. albicans* is a natural commensal of humans, capable of colonizing virtually all anatomical sites (Figure 1). This fungus can switch from harmless commensal to pathogenic growth and thereby cause a spectrum of pathologies, ranging from mucosal to life-threatening systemic infections, collectively termed candidiasis. It is imperative to distinguish the difference between commensal and invasive virulent growth; invasion is distinct from superficial colonization as the former is accompanied by inflammatory signals, resulting from the activated immune response. Clinical cases presenting *C. albicans* infections of the urogenital tract [1,2,3,4,5,6,7,8], kidney [1,7,9,10,11,12,13], liver [14,15], lungs [16,17,18], spleen [19,20] and even the heart [21,22,23,24,25,26] have been reported. In rare cases, mostly in neonates, *C. albicans* can traverse the blood–brain barrier, resulting in infections of the brain [27,28,29,30]. These observations indicate that *C. albicans* can successfully establish and grow in different host niches. Consequently, *C. albicans* must possess the means to successfully adapt in order to obtain and use a wide range of host-derived nutrients. Given their opportunistic character, the question remains open as to how *C. albicans* cells fine-tune their nutrient acquisition machineries to support commensal and pathogenic growth under apparently disparate environmental conditions.

*C. albicans* requires a source of nitrogen to synthesize proteins needed to carry out necessary cellular functions and to generate nucleotides for DNA and RNA synthesis. There is a plethora of nitrogen sources that *C. albicans* can theoretically utilize in the host, for example, amino acids, urea, peptides, proteins and N-acetyl glucosamine (GlcNAc) and even ammonia. Of the nitrogen sources available in the host, amino acids are preferred as they can be easily assimilated and used as both nitrogen and carbon sources [31,32]. Most of the current understanding of nutrient assimilation in *C. albicans* is inferred from extensive studies in the non-pathogenic yeast *Saccharomyces cerevisiae*. However, although many of the regulatory mechanisms operating between the two species are conserved, it is becoming clear that substantial differences exist, likely reflecting the different environments in which these fungi evolved. Although *S. cerevisiae* is readily found freely in nature, *C. albicans* has evolved in close association with mammalian hosts as a commensal. Furthermore, although *S. cerevisiae* prioritizes the ability to ferment sugar even in the presence of oxygen, *C. albicans* relies on mitochondrial oxidative phosphorylation to generate the energy necessary to survive in hosts. Aside from being able to thrive better at higher temperatures, i.e., 37 °C, a striking and important difference between *C. albicans* and *S. cerevisiae* is that *C. albicans* possesses mitochondria with all four proton-pumping and energy-conserving complexes of the respiratory chain, including NADH dehydrogenase (Complex I). In *S. cerevisiae* mitochondria, NADH is oxidized by matrix NADH dehydrogenases that are not coupled to energy-conserving proton-pumping mechanisms; hence, the oxidation of NADH in yeast does not directly contribute to ATP production [33].

The clear differences in metabolism between the established yeast model and the fungal pathogen *C. albicans* need to be considered when analyzing its virulence properties. In this review we focus on amino acid sensing and metabolism with an emphasis on proline catabolism. We begin by introducing some basic concepts regarding nitrogen source utilization and assimilation and then present a more focused discussion regarding amino acid metabolism, the generation of ammonia and associated consequences, and the central role of mitochondria in the production of energy for virulent growth.

## 2. Amino Acids as Nitrogen Sources in Host Environments

**Figure 2:**
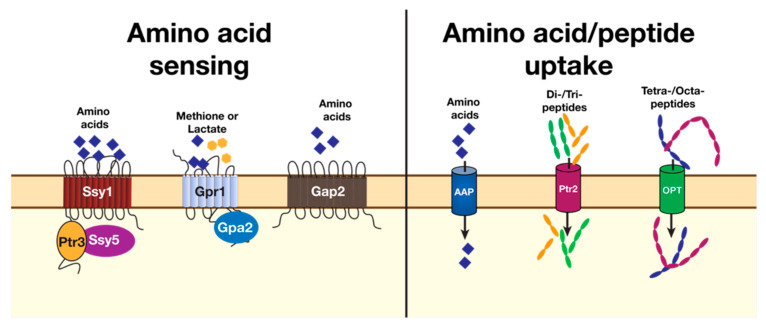
**Amino acid sensors and transporters localized at the plasma membrane of *C. albicans***. (**Left**) the SPS sensor responds to the presence of extracellular amino acids. The main SPS sensor is composed of Ssy1, a plasma membrane-bound receptor homologous to amino acid permeases but without a capacity to transport amino acids; Ptr3, a scaffold protein that mediates intracomplex protein–protein interactions; and Ssy5, an endoprotease that proteolytically activates downstream transcription factors. The G-protein-coupled receptor 1 (Gpr1), together with intracellular cognate protein Gpa2, has been implicated in both amino acid (methionine) and lactate sensing. Gap2 is an ortholog of *S. cerevisiae* Gap1, which is thought to function as a transceptor, i.e., a functional transporter capable of initiating downstream signaling events independently of transport. (**Right**) uptake of extracellular amino acids is facilitated by a number of genetically distinct amino acid permeases (AAP) that have either broad or narrow substrate specificities. Amino acids can also enter the cell as oligopeptides taken up by Ptr2 for di-/tri-peptides, or a family of oligopeptide transporters (OPT) for oligopeptides comprising between 4 and 8 residues.

As an opportunistic pathogen, *C. albicans* can sense a multitude of environmental signals, including changes in the availability of diverse nitrogen sources, including amino acids. The signaling pathways that are induced regulate the activity of downstream effector transcription factors that engage programs of gene expression, some that are required for virulent growth (reviewed in [34,35]). A limited number of amino acid sensors have been characterized in *C. albicans* (Figure 2), the best understood being the plasma membrane-localized SPS sensor of extracellular amino acids [36,37,38]. Strains lacking a functional SPS sensor have a diminished capacity to take up amino acids, do not filament in the presence of serum and are less virulent during systemic infection in mice. These results provide a clear example of how an ordinary basic physiological process, such as nitrogen (amino acid) acquisition, can become an “accidental” but important virulence trait of an opportunistic human pathogen. Additional sensors of external amino acids in *C. albicans* have been reported, such as Gpr1, a G-protein-coupled receptor proposed to sense methionine [39], and Gap2, a general amino acid transporter that is the functional ortholog of Gap1 in *S. cerevisiae* (ScGap1) that could function as a transceptor [40]. Proteins that sense the availability of other well-characterized nitrogen sources, e.g., Mep2 that responds to ammonium [41], can also provide important regulatory signals governing amino acid use. Although *C. albicans* cells have been shown to respond to the presence of GlcNAc [42,43] and urea [44], active sensing mechanisms for these nitrogen sources have not been described. Nitrogen-containing compounds (amino acids) are taken up from the extracellular environment through a number of distinct transporters localized at the plasma membrane (Figure 2). Activation of the SPS sensor enhances the capacity of cells to take up and assimilate diverse nitrogen substances. This is accomplished as the signals derived from the activated SPS sensor induce the expression of several genes encoding amino acid permeases, secreted aspartyl protease *SAP2*, peptide and oligopeptide transport proteins [36,45,46].

## 3. Nitrogen Catabolite Repression (NCR)

In addition to sensing the availability of extracellular sources of nitrogen, yeast cells can gauge the quality of internalized sources of nitrogen and respond appropriately to adjust metabolism. Nitrogen catabolite repression (NCR) is a supra-pathway that controls nitrogen source utilization through the repression of genes required for the utilization of secondary nitrogen sources when preferred ones are available. Most of the assumptions with respect to NCR in *C. albicans* are derived from extensive studies examining NCR in *S. cerevisiae*. Understanding the differences between these organisms is essential to accurately describing the hierarchy of nitrogen source assimilation and use by *C. albicans* during pathogenic growth.

**Figure 3:**
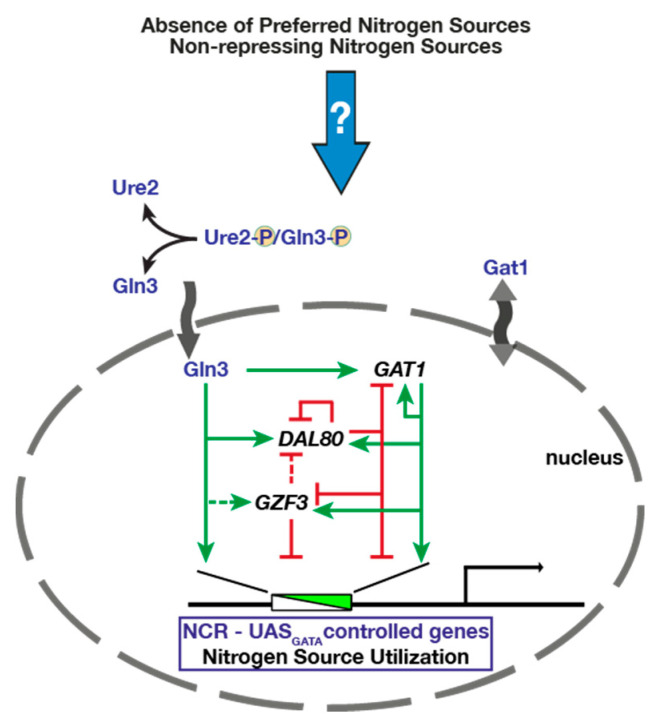
**Schematic diagram of nitrogen catabolite repression (NCR) in yeast** (adapted from Ljungdahl and Daignan-Fornier, 2012 [47]). Gln3 and Gat1 are two positive GATA effectors of NCR that are normally excluded from the nucleus under preferred nitrogen replete conditions. Nuclear exclusion is thought to occur via the interaction of Gln3 with the phosphorylated version of the Ure2 protein.

In *S. cerevisiae*, NCR is controlled by four GATA transcription factors: Gln3, Gat1, Dal80 and Gzf3, all of which possess zinc-finger DNA-binding motifs that recognize a conserved GATAAG consensus sequence present in the promoters of target genes (reviewed in [47]) (Figure 3). Gln3 and Gat1 act as positive regulators of gene expression, whereas Dal80 and Gzf3 act in a negative manner to repress target gene expression. The ability of the GATA factors to compete for binding GATAAG sequences is influenced by nitrogen source availability and is even modulated by events within the nucleus [48,49]. In the presence of preferred nitrogen sources, such as ammonium and certain amino acids, Gln3 and Gat1 are tethered in the cytosol, restricting their translocation into the nucleus. For Gln3, nuclear exclusion is maintained by binding to the phosphorylated species of its interacting partner, Ure2. Gln3 and likely Gat1 can be phosphorylated, but the phosphorylation status of Gln3 does not affect its capacity to bind Ure2. Gln3 targets to and is retained in the nucleus in cells carrying deletion or mutationally inactivated alleles of Ure2, resulting in the constitutive expression of NCR-sensitive genes. Unlike Gln3, Gat1 is not entirely dependent on Ure2 for retention in the cytosol, and therefore other regulatory components apparently contribute to controlling Gat1 movement and inducing activity. Contrary to Gln3 and Gat1, Dal80 and Gzf3 are constitutively localized in the nucleus. Furthermore, in contrast to *GLN3*, *GAT1*, *GZF3* and *DAL80* are expressed under the control of promoters containing multiple GATAAG sequences, placing their expression under NCR [50,51,52,53]. Consequently, these factors participate in regulating each other’s expression (cross-regulation) and in certain instances exhibit partial autogenous regulation [48,52,53,54] (Figure 3).

NCR has been described in *C. albicans*; however, the information available is limited to studies using strains lacking Gln3 and/or Gat1 [55,56,57]. Strains lacking either or both of these GATA transcription factors are unable to efficiently utilize a number of alternative nitrogen sources. This has been shown to be linked to the lack of derepression of genes necessary for their catabolism. Results from the Fonzi laboratory have shown that Gln3 and Gat1 appear to exert both specific and overlapping functions, depending on the available nitrogen sources [56]. Certain amino acids traditionally classified as poor, such as proline in *S. cerevisiae* [47,58], are readily utilized by *C. albicans* lacking Gln3 and Gat1, clearly indicating that proline utilization is not subject to tight NCR control [56]. Consistently, recent work in our group and others have shown that enzymes of the proline catabolic pathway (e.g., Put1 and Put2) can be induced in the presence of preferred nitrogen sources (e.g., ammonium or amino acids) [59,60,61] and even in a strain lacking Gln3 and Gat1 [59]. In addition, the gene encoding glutamate dehydrogenase (*GDH2*), a key player in central nitrogen metabolism, is robustly expressed when there is an abundance of preferred nitrogen sources such as ammonium and amino acids, indicating that its expression is independent of NCR [61]. This latter finding is in striking contrast to *S. cerevisiae*, with its *GDH2* subject to tight NCR control (reviewed in [47]).

These clear differences between *C. albicans* and *S. cerevisiae* are not trivial, and clearly reflect divergent evolutionary trajectories and the need for *C. albicans* to rapidly respond to distinct host environments. For example, as *C. albicans* cells breach epithelial barriers and reach the blood, they are exposed to high concentrations of amino acids, a condition that likely represses NCR-controlled genes, including those required for the assimilation of nitrogen derived from the degradation of host proteins. The transcription factor *STP1* is NCR-controlled and under these conditions is not expressed, which limits the expression of the Stp1-dependent secreted protease Sap2 and oligopeptide transporters.

## 4. Extracellular Amino Acid Sensing and Uptake—The SPS Sensing System

**Figure 4:**
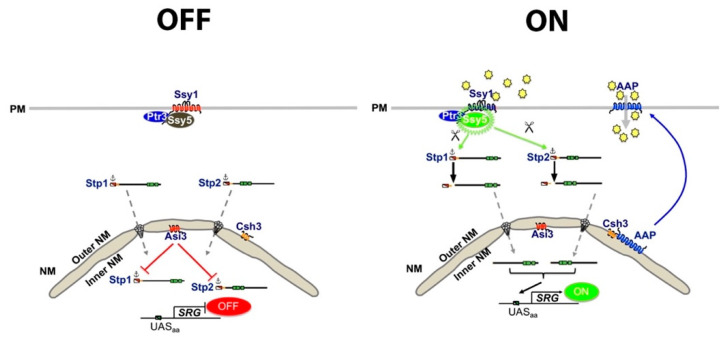
**The SPS sensing system of *C. albicans*.** Ssy1 is the primary amino acid sensor that functions with the scaffold protein Ptr3 and the protease Ssy5 as a multimeric receptor complex. Stp1 and Stp2 are the effector transcription factors of this pathway. ((**Left panel**), OFF state) In the absence of extracellular amino acids, Stp1 and Stp2 are produced as latent cytoplasmic precursors that are retained in the cytosol due to N-terminal regulatory domains that possess both a cytoplasmic retention motif and a nuclear degron, the latter recognized by the E3-ubiquitin ligase, Asi3. ((**Right panel**), ON state) In the presence of amino acids, Ssy1 is stabilized in a signaling conformation, which initiates downstream events, resulting in the activation of the Ssy5 protease. Activated Ssy5 endoproteolytically cleaves the N-terminal regulatory domains of Stp1 and Stp2. The shorter, cleaved forms efficiently translocate into the nucleus, where they bind upstream activating sequences (UASaa) and induce the expression of SPS-regulated genes (SRG). Importantly, Stp1 and Stp2 induce divergent subsets of genes, and *STP1* expression is under NCR control; *STP1* is repressed in cells grown in the presence of millimolar concentrations of amino acids, whereas Stp2 is constitutively expressed. Activated Stp2 induces the expression of amino acid permease (AAP) genes. AAPs are translated and initially inserted in the ER membrane, where they require the assistance of the ER-membrane-localized chaperone Csh3, the ortholog of yeast Shr3, to attain native structures. In the absence of Csh3, AAPs aggregate and are retained in the ER. Activated Stp1 derepresses the expression of *SAP2*, a secreted protease, and multiple oligopeptide transporter genes that facilitate peptide uptake. Stp1 triggers responses required for host protein utilization, whereas Stp2 induces amino acid utilization.

The plasma-membrane-localized SPS (Ssy1-Ptr3-Ssy5) sensor of *C. albicans* has been characterized [36,38,59,62]. The SPS sensor enables cells to sense and respond to the presence of extracellular amino acids (Figure 4). Again, progress has largely been guided by ongoing studies using *S. cerevisiae* as a model. In *S. cerevisiae,* the SPS signaling pathway controls the expression of a distinct set of amino acid permease (AAP) genes encoding transporters catalyzing proton-driven amino acid uptake. Two homologous effector transcription factors, Stp1 and Stp2, are synthesized as inactive precursors that localize to the cytoplasm due to N-terminal regulatory domains. The regulatory domains possess cytoplasmic retention and nuclear degron motifs, both of which are required to maintain the “off-state” of SPS-sensor-regulated gene expression. The cytoplasmic retention motifs prevent these factors from efficiently entering the nucleus, and the degron motif targets the low levels of full-length Stp1 and Stp2 that escape cytoplasmic retention for degradation by means of a novel inner-nuclear-membrane-associated degradation (INMAD) pathway. The INMAD pathway is defined by the E3 ubiquitin ligase Asi complex (Asi1-Asi2-Asi3) [63,64,65]. Extracellular amino acids activate the SPS sensor by binding to the receptor component Ssy1, which undergoes a conformational change that activates the Ssy5 protease in a Ptr3-dependent manner: Ptr3 functions as a scaffold that mediates intracomplex protein–protein interactions. Activated Ssy5 cleaves the N-terminal regulatory domains of Stp1 and Stp2, a processing event that enables the cleaved factors, lacking cytoplasmic retention and degron motifs, to efficiently translocate to the nucleus and bind to upstream activating sequences (UASaa) in the promoters of AAP genes. AAPs are co-translationally inserted into the endoplasmic reticulum (ER) membrane, contiguous with the outer nuclear membrane. The movement of AAPs to the PM requires the ER membrane-localized chaperone Shr3, which facilitates their folding and packaging into ER-derived secretory vesicles, a requisite for their functional expression [66,67]. The SPS sensing system enables amino acids to induce their own uptake.

Orthologs of the SPS sensing system are present in *C. albicans* ([36,37,38,59,62]; reviewed in [35]) (Figure 4). There is, however, a major difference. In contrast to *S. cerevisiae*, Stp1 and Stp2 in *C. albicans* activate different sets of genes that express proteins facilitating the assimilation of distinct external nitrogen sources [36,62]. Stp1 regulates the expression of *SAP2*, encoding the major and broad-spectrum secreted aspartyl proteinase (Sap) and multiple oligopeptide transporters (Opts) [36]. *STP1* expression is subject to NCR and controlled by the GATA transcription factors Gln3 and Gat1 ([68], reviewed in [69]). Accordingly, *STP1* expression is repressed when preferred nitrogen sources, i.e., ammonium sulfate and amino acids, are available and is derepressed when these nitrogen sources become limiting or absent. *STP2* is constitutively expressed and functions analogously to Stp1/Stp2 in *S. cerevisiae* and derepresses the expression of multiple amino acid permeases. *C. albicans* strains lacking either Ssy1 or Csh3, the latter an ortholog of yeast Shr3, fail to efficiently respond to the presence of extracellular amino acids and have impaired an capacity to filament in amino acid-based media [37,38]. Not all amino acids activate the SPS sensor, as can be observed by monitoring the proteolytic processing of Stp2 [36,59]. The capacity to activate the sensor and induce Stp2 processing is limited to a subset of amino acids; the presence of glutamine and arginine leads to robust SPS sensor activation. Stp2 processing is observed 5 min post-induction, indicating that the SPS sensing system rapidly responds to the presence of extracellular amino acids.

Additional sensing systems in *C. albicans*, capable of transmitting signals regarding extracellular amino acid availability, have been reported. The G-protein-coupled receptor (Gpr1) has been proposed to sense extracellular methionine [70] or even glucose, similarly to *S. cerevisiae* [71]. The idea that methionine is the primary activating ligand for Gpr1 stems from the fact that the addition of methionine could trigger the rapid internalization of Gpr1 in a manner consistent with ligand-mediated receptor internalization ([70]; reviewed in [72]). More recently, however, lactate has been proposed to be the primary activating ligand for Gpr1 [73]. Hence, the role of Gpr1 in amino-acid-induced morphogenesis remains to be defined. What is known is that ligand activation of Gpr1 stimulates GTP-GDP exchange in its effector Gα protein Gpa2; the active GTP-bound form of Gpa2 subsequently binds to the Gα-binding domain in the N-terminal region of the adenylate cyclase Cyr1, leading to enhanced cAMP production (reviewed in [74,75]). Null mutations of the *GPR1* or *GPA2* in *C. albicans* diminish filamentous growth on solid media, and consistently, filamentation can be restored via the addition of exogenous cAMP [71]. Interestingly, although Gpr1 and Gpa2 were initially characterized on the basis of increased cAMP synthesis in response to glucose, deletions of *GPR1* or *GPA2* do not affect glucose-induced cAMP signaling, and cells remain responsive to methionine and proline [39,70]. Furthermore, the levels of cAMP in *gpr1*-null mutants spike in response to serum or large amounts of glucose (100 mM, or 1.8%), suggesting that Cyr1 can be activated by Gpr1-independent processes [70].

## 5. Amino Acids from Proteins and Peptides

Although free amino acids are preferred, as they can be rapidly utilized as both carbon and nitrogen sources, the bulk of amino acids in hosts are typically fixed in proteins, e.g., the extracellular matrix proteins collagen and mucin. Consequently, extracellular proteolytic enzymes are required to cleave host proteins to release amino acids and peptide fragments that can subsequently be taken up by the cell. It is important to note that the breakdown of host proteins that occurs at sites of infection can be due to proteases secreted either from the fungal or host cells [76,77]. In vitro, *C. albicans* can acquire peptides and amino acids derived from extracellular proteins, e.g., albumin, collagen and mucin. This requires the expression of secreted aspartyl proteases (Saps) [78,79,80] or the activity of matrix metalloproteinases (MMPs) [81,82]. The host can also trigger the proteolytic degradation of tissues as observed in some pathological conditions such as cancer [83] or sarcopenia (muscle wasting) [84]. Once internalized, peptides are then degraded to amino acids through the activity of several intracellular proteases, e.g., metallopeptidase, dipeptidase, carboxypetidases and serine proteases, which liberate free amino acids [85,86,87,88]. The induced expression of some of these enzymes is complex and depends on the release from strict regulatory processes, including NCR [68]. Although the overall effect on virulence remains to be clarified, the discovery of the fungal toxin candidalysin is important as it can also trigger the release of free amino acids by contributing to the lysis of host cells [89,90,91].

## 6. Amino Acid Metabolism in *C. albicans*

**Figure 5:**
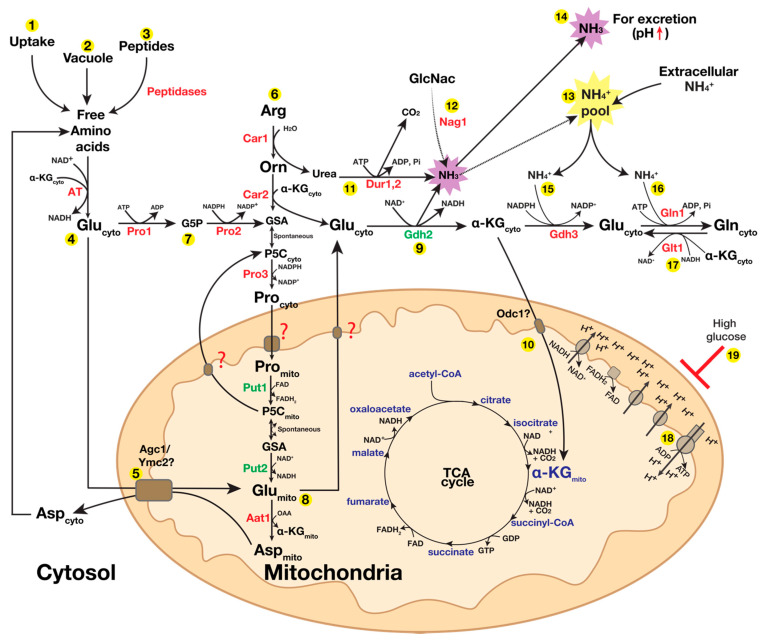
**Nitrogen utilization in *C. albicans*.** Most free amino acids, obtained from extracellular uptake (**1**), vacuolar release (**2**) or the degradation of small peptides by intracellular peptidases (**3**), are deaminated by specific aminotransferases (ATs) using α-ketoglutarate as the amino group acceptor, forming glutamate. Many ATs are found in the cytosol and there is an abundant supply of glutamate in the cytosol (Glu_cyto_) (**4**). Glutamate can be imported into the mitochondria by transporters, such as Agc1 (**5**). Arginine and proline are converted to glutamate via the proline catabolic pathway (**6**). In the cytosolic portion of this pathway, arginine is converted to ornithine and urea by arginase (Car1). Ornithine is rapidly converted to glutamate semialdehyde (GSA) by ornithine transaminase (Car2), which is non-enzymatically converted to the cyclic Δ-1-pyrroline-5-carboxylate (P5C) and then reduced by P5C reductase (Pro3), generating proline (Pro_cyto_). Proline enters the mitochondria (Pro_mito_) via an unidentified transporter. Pro_mito_ is catabolized to glutamate (Glu_mito_) via the concerted activities of proline dehydrogenase (Put1) and P5C dehydrogenase (Put2). Glutamate produced in the mitochondria (Glu_mito_) (**8**) is thought to exit the mitochondria and become part of Glu_cyto_. Glu_cyto_ is used in the synthesis of proline (**7**); glutamate is first activated to produce glutamate-5-phosphate (G5P) by γ-glutamyl kinase (Pro1), followed by its conversion to GSA/P5C by γ-glutamyl phosphate reductase (Pro2) and is then reduced to proline by P5C reductase (Pro3). Cytosolic glutamate can be converted to α-ketoglutarate by the NAD^+^-dependent glutamate dehydrogenase (Gdh2), which is critical for maintaining the α-ketoglutarate pool in the cytosol (α-KG_cyto_). The reaction catalyzed by Gdh2 generates ammonia as a by-product (**9**). α-ketoglutarate can be transported in and out of mitochondria via putative oxodicarboxylate carriers (e.g., Odc1). The mitochondrial α-KG_mito_ pool is linked to the TCA cycle (**10**). Urea, derived either from arginine or from extracellular uptake, can be converted to ammonia and CO_2_ via urea amidolyase (Dur1,2) (**11**). When grown in the presence of N-acetylglucosamine (GlcNac), ammonia is also produced when glucosamine-6-phosphate is converted to fructose-6-phosphate through glucosamine-6-phosphate isomerase (Nag1) (**12**). As the cytosolic pH is maintained near neutrality (pH~6.5), most ammonia is converted to its protonated form, ammonium (**13**). Free ammonia is membrane-permeable and can readily exit cells, where it contributes to the alkalization of the growth environment, a consequence of its conversion to ammonium (**14**). Ammonium in cells can be reassimilated to generate glutamate by the NADPH-dependent glutamate dehydrogenase (Gdh3), which uses α-ketoglutarate as a substrate (**15**); additionally, ammonium can be reassimilated via glutamine synthetase (Gln1), which catalyzes the conversion of glutamate to glutamine (**16**). Glutamate can also be generated from glutamine and α-ketoglutarate via the NADH-dependent glutamate synthase (Glt1) (**17**). In mitochondria, the NADH/FADH_2_ generated by the TCA cycle and proline catabolism can be oxidized via the electron transport chain (ETC) to generate ATP (**18**). Mitochondrial function and multiple enzymatic activities are repressed in cells grown in the presence of high glucose (≥0.2%, (**19**)). The localization of enzymes shown in green have been experimentally validated in *C. albicans*, whereas those shown in red are based on the localization of their corresponding orthologs in *S. cerevisiae* and the presence or absence of strong mitochondrial pre-sequences in the N-terminals of their respective protein sequences (Candida Genome Database (CGD, http://www.candidagenome.org (accessed on 18 December 2021)) analyzed using the MitoFate tool [92]. The following enzymes are present in the *C. albicans* genome—PRODH = *PUT1* (C5_02600W), P5CDH = *PUT2* (C5_04880C), GDH = *GDH2* (C2_07900W), P5CR = *PRO3* (C4_00240), OAT = *CAR2* (C4_00160C), ARG = *CAR1* (C5_04490C), GK = *PRO1* (CR_10580), GPR = *PRO2* (C3_07220C). *GLN1* (CR_05050W), *GLT1* (C1_06550W), *DUR1,2* (C1_04660W), NADPH-dependent GDH = *GDH3* (C4_06120W), *AAT1* (C2_05250C) and *AAT2* = *AAT21* (CR_07620W).

Many amino acids, derived either from extracellular uptake or oligopeptide/protein degradation or released from the vacuolar compartment, are converted to glutamate in the cytosol via the catalytic activity of distinct aminotransferases (ATs) (Figure 5). Specifically, ATs transfer the α-amino group of an amino acid to α-ketoglutarate (α-KG; 2-oxoglutarate), resulting in the formation of glutamate. ATs are defined by the amino acid that serves as the amino group donor. ATs collectively contribute to the cytosolic glutamate pool (Glu_cyto_). Examples include aspartate aminotransferase (Aat1, EC 2.6.1.1), which transfers the α-amino group of aspartate to α-KG, resulting in glutamate and oxaloacetate; ornithine transaminase (Car2; EC 2.6.1.13), which uses ornithine, forming glutamate and glutamyl-5-semialdehyde; alanine transaminase (Alt1; EC 2.6.1.2), which uses alanine, forming glutamate and pyruvate; and glutamate synthase (Glt1; EC 1.4.1.14), which uses glutamine, forming glutamate.

Glutamate is enzymatically converted to α-KG via oxidative deamination, catalyzed by NAD^+^-dependent glutamate dehydrogenase (Gdh2; EC 1.4.1.2), yielding ammonia (NH_3_) and reduced NADH [61,93]. At a physiologically relevant pH, ammonia is protonated to ammonium (NH_4_^+^). In mammalian cells, glutamate dehydrogenase is localized in the mitochondria, whereas in *S. cerevisiae,* there is a lack of consensus regarding its localization; Gdh2 has been reported to be a mitochondrial component [94,95] and alternatively a cytosolic component [96,97,98]. The bulk of ammonia produced by *C. albicans* during growth on amino acids as a sole nitrogen and carbon source comes from the reaction catalyzed by Gdh2 [61]. Contrary to the initial publication, now corrected [61], Gdh2 in *C. albicans* is clearly cytoplasmic. The correct assignment of Gdh2 as a cytoplasmic component is key to understanding its role in cellular energy production, as the reaction reduces NAD^+^, forming NADH. The ammonium pool in the cytosol generated primarily from Gdh2 activity and possibly from the import of extracellular ammonium via the ammonium transporters Mep1 and Mep2 [57,99,100] can be assimilated by two key anabolic reactions catalyzed by the NADPH-dependent glutamate dehydrogenase (Gdh3, EC 1.4.1.4; Gdh1 in *S. cerevisiae)* and glutamine synthetase (Gln1; EC 6.3.1.2) (Figure 5). Gdh3 catalyzes the synthesis of glutamate from α-KG and ammonium [93], whereas Gln1 catalyzes the synthesis of glutamine from glutamate and ammonium in an ATP-dependent reaction [93]. In *S. cerevisiae*, cytosolic glutamate can be imported to the mitochondrial matrix via transporters localized at the inner mitochondrial membrane, such as Agc1 [101,102,103,104] or Ymc2 [104]. Putative orthologs of these proteins exist in *C. albicans* (see CGD, http://www.candidagenome.org (accessed on 19 December 2021); C1_13400C for Agc1 and C4_02080W for Ymc2). Furthermore, in *S. cerevisiae*, cytosolic α-KG can be imported into the mitochondria through oxodicarboxylate carriers that exists in two isoforms, Odc1 and Odc2; Odc1 is used during respiration and its expression is subject to glucose repression, whereas Odc2 is the predominant isoform under non-respiratory conditions [105,106]. The *C. albicans* genome has a putative ortholog for Odc1 (CR_05480W).

In addition to the cytoplasmic glutamate pool (Glu_cyto_), a significant fraction of the intracellular glutamate is generated in the mitochondria (Glu_mito_) via the proline catabolic pathway. In eukaryotes, the four-electron catabolic conversion of proline to glutamate is carried out through the successive actions of proline dehydrogenase (PRODH; EC 1.5.5.2) and Δ1-pyrroline-5-carboxylate (P5C) dehydrogenase (P5CDH; EC 1.2.1.88). PRODH and P5CDH are highly conserved enzymes throughout eukaryotes and bacteria (reviewed in [107,108,109,110]). In *C. albicans*, PRODH and P5CDH are called Put1 and Put2, respectively, and both are nuclear-encoded mitochondrial proteins [59,61]; in most eukaryotes, PRODH is associated with the inner mitochondrial membrane and is connected to complex II of the electron transport chain (ETC; reviewed in [110]). Cytosolic proline (Pro_cyto_), derived from uptake, biosynthetic reactions or from the catabolism of arginine or ornithine [59], is imported into the mitochondria (Pro_mito_) via a still-unidentified mitochondrial transporter. PRODH then transfers two electrons from proline to FAD to generate P5C and the reduced flavin cofactor (FADH_2_). P5C tautomerizes spontaneously in a non-enzymatic reaction, forming glutamic-γ-semialdehyde (GSA). The prevailing pH strongly affects the equilibrium between P5C and GSA; P5C formation is favored when the pH is >6.5. P5CDH then catalyzes the oxidation of GSA to glutamate, reducing NAD^+^ to NADH. When high levels of proline are available and catabolized, P5C can accumulate in the mitochondria and exert a toxic effect [111,112,113]. The reduced cofactors FADH_2_ and NADH, generated by proline catabolism, are oxidized by the ETC of mitochondria to power ATP generation. Since Gdh2 catalyzes the conversion of glutamate to α-KG in the cytosol and that Gdh2-dependent alkalization is tightly linked to mitochondrial function [61], it is highly likely that glutamate resulting from proline catabolism (Glu_mito_) is able to exit the mitochondria. To date, a dedicated glutamate transporter capable of exporting glutamate out of the mitochondria has yet to be identified. In yeast, mitochondrial glutamate is converted to aspartate by the mitochondrial aspartate aminotransferase (Aat1; EC 2.6.1.1), and aspartate exits the mitochondria via the Agc1 antiporter. The relevance of this transporter with respect to the export of glutamate is not clear, as the antiporter mechanism transports aspartate out and glutamate in. Aspartate in the cytosol can be converted back to glutamate via the cytosolic aspartate aminotransferase (Aat2; EC 2.6.1.1). The *C. albicans* genome has putative orthologs of Aat1 (*AAT1*; C2_05250C) and Aat2 (*AAT21*; CR_07620W). In yeast, cytosolic glutamate is used in the biosynthesis of several amino acids, including proline; 85% of the total cellular nitrogen is incorporated via the amino nitrogen of glutamate, and the remaining 15% is derived from the amide nitrogen of glutamine [114].

## 7. Mitochondrial Metabolism Is Sensitive to Glucose Availability in *C. albicans*

Amino acid metabolism can be directly or indirectly regulated by glucose. Direct control is exerted by Mig1 and Mig2, well-studied factors that bind promoters and repress transcription when glucose is abundant [115]. Indirectly, glucose can negatively and pleiotropically regulate amino acid metabolism by controlling the function of mitochondria. For example, we and others have shown that *C. albicans* mitochondrial activity can be downregulated by glucose in a manner similar to *S. cerevisiae,* albeit to a lesser extent [59,116]. Our data indicate that the repressing effect of glucose is clearly evident in *C. albicans* at 0.2% or higher [59], and more sensitive transcriptomic studies have noted effects of 0.01% glucose, a very low level of glucose [116]. The more pronounced repressive effect of glucose on mitochondrial respiration in *S. cerevisiae* is likely due to the limited capacity to oxidize NADH when glycolytic flux is high [117]. This is expected to be similar in *C. albicans*; however, since *C. albicans* has a functional complex I with a higher capacity to oxidize NADH, the threshold level for glucose’s repression of mitochondrial respiration is higher than that in *S. cerevisiae*. Consistently, we observed that the level of ATP is higher in 0.2% glucose than in 2% glucose [59]. Consistently with the model that ATP-dependent Ras1 activation drives filamentous growth [118], filamentation is more robust when glucose is <0.2% [39,59]. The lower ATP level observed for cells grown in the presence of 1% glycerol is likely due to the lower levels of reduced NADH (low NADH/NAD^+^ ratio) that can be oxidized to generate the membrane potential needed to generate ATP [59].

The pleiotropic effect of glucose on mitochondrial activity and amino acid catabolism is nicely illustrated by arginine catabolism. Arginine catabolism occurs in a bifurcated manner that generates two products that are independently catabolized either in the cytosol (urea) or mitochondria (proline). When glucose is absent, the proline catabolic pathway becomes essential for arginine catabolism as cells lacking *PUT1* or *PUT2* failed to grow in the presence of arginine as the sole nitrogen and carbon source, whereas cells lacking *DUR1,2* grew [59]. However, when high glucose was added as the main carbon source, the *put1*−/− defect was rescued as the metabolism shifted from pure respiratory to mixed types of growth (respiratory/fermentative), shifting the metabolic burden of nitrogen assimilation to the cytosolic Dur1,2. Strikingly, the enzymes required to catabolize proline were still expressed; however, since the mitochondria were repressed by high glucose levels, they were unable to carry out their catabolic functions of converting proline to glutamate.

## 8. Ammonia Generation and Excretion

**Figure 6:**
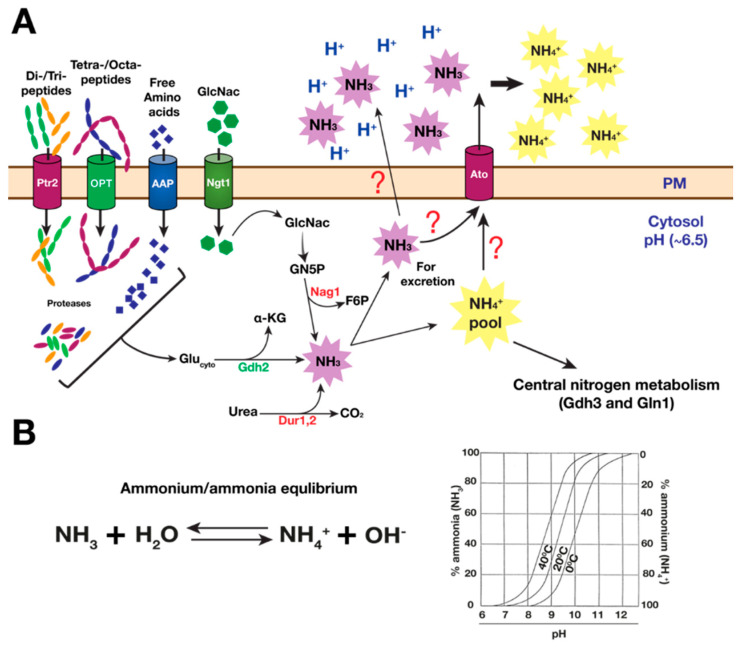
**Ammonia extrusion in *C. albicans*.** (**A**) When *C. albicans* utilizes amino acids or N-acetylglucosamine (GlcNac) as nitrogen sources for growth, ammonia is produced, promoting the alkalization of the extracellular pH. Amino acids can be either free or derived internally from the proteolytic degradation of oligopeptides, which are then converted to glutamate either in the cytosol (by specific aminotransferases) or mitochondria (proline catabolism), creating a glutamate pool in the cytosol (Glu_cyto_). The NAD^+^-dependent glutamate dehydrogenase (Gdh2) catalyzes the conversion of Glu_cyto_ to α-ketoglutarate, releasing ammonia in the process. Urea generated via arginine catabolism (or from the extracellular environment) can also be degraded to ammonia via the urea amidolyase (Dur1,2) enzyme. The deamination of GlcNac is dependent on glucosamine-6-phosphate isomerase (Nag1), which catalyzes the conversion of glucosamine-5-phosphate (GN5P) to fructose 6-phosphate (F6P). Excess ammonia produced in the cytosol must be removed in order to avoid its toxic effects; due to the cytosolic pH of around 6.5, most of the free ammonia (NH_3_) is converted to ammonium (NH_4_^+^) (see graph below), which can be directly assimilated via central nitrogen metabolism (i.e., Gdh3 and Gln1), whereas a small fraction is released into the environment, where it could neutralize the acidic pH, producing ammonium (NH_4_^+^). Whether ammonia (or even ammonium) is exported through simple diffusion or via exporters (Ato) requires further study. (**B**) At the normal cytosolic pH of ≈6.5, ammonia (NH_3_) is converted to ammonium (NH_4_^+^). We present a plot showing the relative concentrations of NH_3_ and NH_4_^+^ in aqueous solution based on pH and temperature. Image adapted and redrawn from Huang, J; *Handbook of Environmental Engineering*) [121]. The ratio of NH_4_^+^ to NH_3_ in this equilibrium is highly pH-dependent. At low acidic pH, the ammonium form (NH_4_^+^) dominates. As the pH increases, the ammonia (NH_3_) form also increases, and the proportion becomes equal at the pKa value. Higher temperatures favor the NH_3_ gas side of the equilibrium balance with NH_4_^+^.

In the human body, amino acids are an abundant source of nitrogen for *C. albicans.* However, the utilization of amino acids in excess of the amount necessary to support growth and basic cellular functions must be controlled due to the accumulation of ammonia as a metabolic byproduct. Excess ammonia is toxic to cells. When cells are grown using amino acids as energy sources, excess ammonia exits into the extracellular medium, resulting in environmental alkalization (Figure 6A). Interestingly, this capacity to increase environmental pH via ammonia extrusion is believed to support the pathogenic growth of fungal pathogens such as *C. albicans* in certain acidic microenvironments, e.g., the phagosome of macrophages (reviewed in [119,120]).

It has been proposed that ammonia derived from amino acid catabolism enables *C. albicans* cells to neutralize the acidic luminal pH of the phagosome, reducing the activities of hydrolytic enzymes with low pH optima and inducing *C. albicans* to switch morphologies, resulting in hyphal growth, thus facilitating macrophage evasion [31]. This model was largely premised on studies using a strain lacking *STP2* (*stp2*Δ/Δ), the SPS transcription factor that positively regulates the expression of amino acid permeases required for amino acid uptake [31,32]. Strains carrying *stp2*Δ/Δ exhibit defects in both environmental alkalization and the capacity to escape the phagosome of the engulfing macrophage [31,32]. This model assigned the critical ammonia-generating event to the urea amidolyase (Dur1,2), which catalyzes the conversion of urea to ammonia and CO_2_, as cells lacking this enzyme (*dur1,2*Δ/Δ) show alkalization defects when cells are grown in media with high glucose [32]. However, Dur1,2 has only been linked to ammonia generation in the presence of its substrate urea, and *DUR1,2* expression is under tight NCR control [44,56]. Thus, it is unlikely that Dur1,2 contributes to the alkalization of a growth medium with abundant preferred nitrogen sources such as amino acids and even ammonium sulfate.

There is mounting evidence that alkalization of the phagosomal compartment is not requisite for *C. albicans* cells to evade macrophages. Results obtained using dual-wavelength ratiometric fluorescence imaging to quantify the pH in the phagosome revealed that increased phagosomal pH is the consequence of elongating hyphal cells, physically stretching the phagosomal membrane, causing transient leaks [122]. The induction of hyphal growth was observed to precede alkalization. In addition, the proton-pumping activity of V-ATPase exceeds the rate of ammonia extrusion by several orders of magnitude [122]. Recently, we reported that Gdh2, the enzyme that catalyzes the conversion of glutamate to α-KG, is responsible for the bulk of ammonia produced from amino acid metabolism [61]; a *gdh2*-null strain is unable to alkalize a medium containing amino acids as the sole nitrogen and carbon source. Surprisingly, the capacity of *gdh2*−/− mutants to escape the macrophage phagosome or its virulence in murine systemic infection model was not affected, indicating that amino acid-dependent environmental alkalization is not essential for the virulence of *C. albicans*. Consistently with a previous report [122], we observed that viable wildtype cells pre-stained with a pH-sensitive dye (pHrodo), of which the fluorescence intensity varied inversely to pH, were retained in acidic phagosomes, and this observation is was even when a high MOI (more ammonia-extruding cells) was used [61].

In *C. albicans*, Gdh2 is a cytosolic component [61] and its expression is independent of NCR; Gdh2 is well expressed in cells grown in a medium with high levels of amino acids, even when supplemented with high levels of ammonium sulfate [61]. Consistently, a strain lacking *GLN3* and *GAT1*, which encode for the GATA transcription factors activating NCR-sensitive genes, remain alkalization-competent (our unpublished data). Despite its cytosolic localization, Gdh2-dependent alkalization is tightly linked to mitochondrial function as acute inhibition of the mitochondria with a sublethal dose of antimycin, a potent respiratory complex III inhibitor, virtually abolished alkalization in wildtype cells even when a very high starting cell density was used (OD_600_ ≈ 5) [61]. Since proline catabolism is a major source of glutamate in the mitochondria and since this alkalization is partially dependent on proline catabolism [61], it is likely that pharmacological inhibition of the mitochondria pleiotropically prevented either the generation or export of mitochondrial glutamate. In a similar way, the inability of *C. albicans* to alkalinize the extracellular environment when grown in the presence of high glucose (2%) is likely due to the capacity of glucose to pleiotropically inhibit or downregulate mitochondrial function [59,116]. Gdh2 levels appear to be regulated by pH as the protein levels decrease as the pH of the growth medium approaches neutrality, which is consistent with the observed dependency of alkalization on the starting cell density [61]. In addition to amino acids, growth on N-acetylglucosamine (GlcNac) can also raise extracellular pH via ammonia extrusion. However, the origin of alkalinizing ammonia is distinct as it is catalyzed by the enzyme glucosamine-6-phosphate isomerase (Nag1), which deaminates glucosamine-5-phosphate (GN5P), converting it to fructose 6-phosphate (F6P) [123].

Regarding the fate of intracellular ammonia, in aqueous solution, ammonia can exist either as a gas (NH_3_, ammonia) or as a cationic (NH_4_^+^, ammonium) species; the ratio (ammonia/ammonium) increases with pH (pKa = 9.25) (Figure 6B). Since the pH of the cytosol in actively growing *C. albicans* wildtype cells is maintained at ~6.5 [124], the protonated form NH_4_^+^ predominates and can be directly assimilated by the NADPH-dependent glutamate dehydrogenase (Gdh3) or glutamine synthetase (Gln1). Due to its being positively charged, ammonium cannot readily diffuse out of cells, but rather requires transporters or channels to traverse the phospholipid bilayer of biomembranes. A small fraction of the total ammonia species exists in the unprotonated form (NH_3_) that can be released into the extracellular space, where it could exert its neutralizing effect by reacting to the hydrogen ions (H^+^), generating ammonium (NH_4_^+^). How ammonia traverses the plasma membrane from the cytosol is unclear, as proposed earlier, because ammonia extrusion requires the ammonia transport outward (Ato) proteins, a family of plasma-membrane-bound proteins thought to facilitate ammonia export [32,125]. Strains lacking *ATO5* (*ato5*Δ/Δ) or a dominant point mutation in *ATO1* (*ATO1^G53D^*) show strong alkalization defects, and consistently, the overexpression of *ATO* genes accelerates alkalization [32,125]. However, it is also known that ammonia (NH_3_) is membrane-permeable and can easily diffuse out of yeast cells [126,127,128]. Whether ammonia (or even ammonium) is exported through simple diffusion or via exporters (Ato) remains to be clarified.

In addition to ammonia extrusion, yeast cells have an alternative mechanism to minimize the toxic effects of ammonia. *S. cerevisiae* can indirectly limit the production of ammonia by excreting cytosolic amino acids such as glutamate to the extracellular space via proteins that belong to the multidrug resistance transporter family that are thought to function as H^+^ antiporters (e.g., Aqr1) [129]. Whether the same amino acid extrusion process, limiting intracellular ammonia production, operate in *C. albicans* is not yet known but a putative *AQR1* homolog has been identified in the *C. albicans* genome (*QDR2*/C3_05570W). Qdr2 may perform the same function, constituting a rudimentary ammonia detoxification mechanism in *C. albicans*.

## 9. Conclusions and Outlook

*C. albicans* is an opportunistic fungal pathogen that is intimately linked to its human hosts. Since *C. albicans* grows in symbiosis with humans, fungal cells must survive and propagate under identical physiological conditions as human cells. The capacity of *C. albicans* to establish persistent infections relies heavily on their capacity to assimilate nutrients in a competitive landscape where both hosts cells and even other members of the microbiome compete for nutrients. Amino acids are among the most versatile nutrients available in the hosts; they can be assimilated as both nitrogen and carbon precursors, transformed to key metabolic intermediates or utilized to modulate extracellular pH via ammonia formation. Although *S. cerevisiae* paved the way for most of our understanding of nutrient assimilation and metabolic processes in yeasts, there are clearly significant differences that exist in *C. albicans* that must be taken into account as they are crucial to our understanding of how this fungal pathogen assimilate nutrients in the host, especially in the context of infectious growth.

Some of the so-called poor or non-preferred nitrogen sources in *S. cerevisiae*, such as proline, are efficiently utilized by *C. albicans*. This observation is in alignment with recent findings that the enzymes required to utilize proline in *C. albicans* are independent of NCR, allowing the unrestricted utilization of proline regardless of whether other nitrogen sources are available [59,60]. Proline constitutes some of the most abundant proteins in humans (e.g., collagen, mucin); thus, given that *C. albicans* possesses a multi-subunit respiratory complex I (NADH dehydrogenase), similar to human cells, it is not surprising if *C. albicans* evolved to prefer this amino acid as an energy source for growth. Interestingly, proline has long been known as one of the most potent inducers of yeast-to-hyphal transitions, a key virulence factor in *C. albicans* [59,130,131,132,133]. We have shown that the induction of morphogenesis occurs via ATP-dependent Ras1 activation [59]. One molecule of proline can be completely oxidized to generate approximately 30 ATP equivalents [134,135], reinforcing the idea that proline is an important energy source for many types of cells, especially under nutrient-limited conditions. We have shown that *C. albicans* growth in the phagosome of the macrophage is dependent on proline catabolism to obtain energy to survive despite a multitude of environmental stresses [59]. The inadvertent replication of our previous data [59] in the corrected paper [61] highlights the idea that proline is sensed by *C. albicans* in the phagosome of macrophages. Our data are also consistent with recent transcriptomic data showing that proline induced the expression of *ICL1*, a gene encoding the key glyoxylate cycle enzyme isocitrate lyase 1 (Icl1), which is known to be derepressed in *C. albicans*, being engulfed by macrophages [60]. Consequently, strains lacking the capacity to utilize proline have defects in escaping the phagosome of macrophages [59]. In terms of environmental alkalization, proline catabolism plays a major role by virtue of glutamate production (Put2 product). In the presence of arginine as the sole nitrogen and carbon source, proline catabolism is essential as it is the primary catabolic route to generate glutamate, which can then be subsequently catabolized by Gdh2 to ammonia and α-KG, a key TCA cycle intermediate. However, in the presence of other amino acids, the proline catabolic pathway becomes less essential for alkalization as other amino acids can be transaminated to generate glutamate (Figure 5). Proline utilization in *C. albicans* provides a clear example of how evolution influences and fine-tunes metabolism, leading to unique capabilities, in this case to the utilization of nutrients in a manner not relevant for other related yeasts. Consequently, a thorough examination of other amino acid catabolic pathways in *C. albicans* is warranted, the premise being that many important regulatory differences may exist, and that these may be specifically linked to the evolution of *C. albicans* within mammalian hosts.

A major challenge to correctly interpret experimental results derived from studies examining nutrient sensing and assimilation in *C. albicans* is understanding how laboratory growth conditions influence the results. Many of the standard laboratory conditions do not reflect the mammalian micro-niches in which *C. albicans* resides. For example, many host–pathogen interaction experiments involving innate immune cells are carried out in cell culture medium (RPMI or DMEM) containing 5–10% fetal bovine serum. These media readily trigger filamentous growth in *C. albicans*, independently of host cell interactions (e.g., with macrophages), resulting in the false impression that certain genes are not important for the survival of *C. albicans* during co-culture with innate immune cells. This is especially crucial when looking at the role of specific genes that are required for nitrogen acquisition. For example, there is a possibility that the importance of certain genes under NCR control will be erroneously dismissed as they are not expressed under nitrogen-replete conditions such as those in cell culture media. In addition, it is common practice to use strains pre-grown in YPD, a complex medium that is high in glucose (2%) and rich in nitrogen (amino acids, peptides), prior to shifting cells to desired experimental test conditions. In humans, the level of blood glucose is maintained within homeostatic limits (0.05–0.1%; 3–5 mM glucose) that are well below the level used in YPD [116]. The dramatic reorientation of metabolism resulting from merely shifting conditions is likely to influence the response, and in many instances may provide a conflicting readout. For example, yeast cells grown in YPD build up an extensive reservoir of amino acids with vacuolar pools during growth in nitrogen-rich conditions [136,137]. This influences nutrient-based signals. Furthermore, many studies have relied on fixed-point microscopy coupled with differential staining to observe and deduce the role of specific mutations on filamentous growth. This approach relies heavily on observing obvious growth defects that may not be readily apparent on strains lacking genes relevant to nutrient acquisition. Although useful information has been obtained, many of these results often reflect “general” rather than “niche-specific” hyphal defects, highlighting the need to identify more suitable laboratory conditions that better mimic mammalian microenvironments.

Although a great deal of information regarding nutrient-induced processes in *C. albicans* is accumulating, there are major gaps in our knowledge with respect to the contribution of the host. The availability of assimilable nitrogen sources, i.e., the abundance of amino acids released as a result of host activities, is often overlooked. The contribution of host-derived activities to the degradation of the extracellular matrix (ECM) during stress due to the proteolytic activities of proteases secreted by different cell types is not fully understood. For example, in people of advanced age, who due to medical advances represent a growing population, often suffer from sarcopenia or muscle wasting. A hallmark of sarcopenia is that the amino acid proline is elevated in the blood, indicating the degradation of structural proteins rich in proline such as collagen [84]. Indeed, the elevation of free amino acids in the blood is linked to other pathological states in humans, including cancer [83,84]. It is likely that amino acid limitation influences the capacity of cancer cells to establish malignant forms of growth; cancer cells have been found to exhibit enhanced rates of amino acid uptake [138]. Furthermore, amino acid metabolism is an important factor during wasting in cancer patients (cachexia) and in aging individuals [139,140]. Clearly, illuminating the entire repertoire of regulatory mechanisms associated with amino acid signaling is crucial to understanding life processes in both healthy and disease states, and studies in *C. albicans* may provide important insights with clear therapeutic applications.
